# Critical scaling of cortical ensembles without cooperative amplification

**DOI:** 10.64898/2026.10.07.757392

**Published:** 2026-10-14

**Authors:** Tiago L. Ribeiro, Raiyyan Siddiqui, Bridgette Gifford, Sinisa Pajevic, Dietmar Plenz

## Abstract

Cortical neurons are sparsely and weakly connected, motivating the long-standing idea that they must act collectively, as cell assemblies, to exert substantial influence on surrounding networks. Yet a few spikes in a single pyramidal neuron can recruit thousands of cortical neurons according to a power law with exponent ∼0.285, consistent with a two-dimensional system near a critical percolation transition. Here we holographically stimulated ensembles of 1–16 layer 2/3 pyramidal neurons in visual cortex of awake mice while imaging hundreds of neighbouring neurons. Across two orders of magnitude in evoked spike count, network responses followed the same scaling law observed for single-neuron perturbations, with no detectable threshold at which co-activation produced additional amplification. Recruitment was sublinear, suppression increased with ensemble size, and ongoing scale-free avalanche dynamics remained stationary. Nevertheless, ensemble identity became increasingly decodable from surrounding activity. Thus, coordinated firing increases representational specificity without escaping the critical dynamics governing propagation: cortical ensembles convey identity without gaining disproportionate causal impact.

